# Natural Flavonoid Nobiletin Attenuates Allergic Asthma via Suppression of STAT3/PI3K‐AKT and Neutrophil Extracellular Traps

**DOI:** 10.1002/fsn3.71083

**Published:** 2025-10-16

**Authors:** Wen He, Yuqing Xu, Lin Zhu, Juzhang Li, Jianfeng Zhang, Qiaozhen Wu

**Affiliations:** ^1^ Department of Respiratory and Critical Care Medicine Suzhou Ninth Hospital Affiliated to Soochow University Suzhou China; ^2^ Central Laboratory Suzhou Ninth Hospital Affiliated to Soochow University Suzhou China

**Keywords:** allergic asthma, neutrophil extracellular traps, nobiletin, PI3K‐AKT signaling pathways, STAT3 signaling pathways

## Abstract

Allergic asthma is classically defined by eosinophilic inflammation and Th2 immune responses. However, neutrophils are increasingly recognized as key contributors, particularly through the release of neutrophil extracellular traps (NETs), which exacerbate airway inflammation by promoting antigen presentation and amplifying Th2 responses. Recent studies have identified an immunoglobulin E (IgE)‐nicotinamide adenine dinucleotide phosphate oxidase 2 (NOX2)‐reactive oxygen species (ROS)‐nitric oxide (NO)‐myeloperoxidase (MPO) axis as a critical NET‐inducing pathway, independent of inducible nitric oxide synthase (iNOS). This study investigated whether nobiletin, a citrus‐derived polymethoxyflavonoid, attenuates allergic airway inflammation by targeting NET formation. Using an ovalbumin (OVA)‐induced murine model of asthma, we assessed the effects of nobiletin on airway pathology, immune cell infiltration, cytokine expression, and signaling pathway activity through multiple cellular and molecular assays. Nobiletin treatment significantly reduced airway inflammation, mucus hypersecretion, and collagen deposition, while it decreased eosinophil and neutrophil accumulation in bronchoalveolar lavage fluid and lung tissue. It also suppressed Th2 cytokines (IL‐4, IL‐5, IL‐13) and proinflammatory mediators (IL‐6, IL‐17A, TNF‐α). Mechanistically, nobiletin inhibited NET formation by blocking PI3K/AKT and STAT3 signaling, with molecular docking confirming its binding to both targets. These findings identify nobiletin as a promising dual‐target therapeutic agent that concurrently modulates Th2‐driven and neutrophil‐mediated inflammation in allergic asthma.

## Graphical abstract

This study demonstrates that the natural flavonoid nobiletin alleviates allergic asthma by suppressing STAT3/PI3K‐AKT signaling pathways and neutrophil extracellular trap formation.

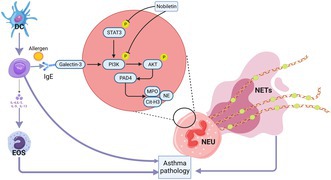

## 1 Introduction

Bronchial asthma is a chronic inflammatory airway disorder characterized by eosinophilia, elevated systemic IgE levels, mucus hypersecretion, and airway hyperresponsiveness. Globally, approximately 262 million people suffer from asthma, with prevalence ranging from 5% to 10% across countries, underscoring its major public health burden (Lommatzsch et al. 2023). While allergic asthma is traditionally associated with eosinophilic inflammation and Th2 responses, growing evidence shows that neutrophils also contribute significantly to disease pathogenesis (Mincham et al. 2024; Radermecker et al. 2018). Neutrophils in allergic asthma exhibit functional plasticity, particularly the ability to release neutrophil extracellular traps (NETs). NETs intensify Th2‐driven pathology by enhancing dendritic cell antigen presentation and stimulating IL‐4, IL‐5, and IL‐13 production (Chacon et al. 2024; Radermecker et al. 2019; Toussaint et al. 2017). Importantly, IgE‐mediated environments prime neutrophils for NETosis via the recently identified immunoglobulin E (IgE)‐nicotinamide dinucleotide phosphate oxidase 2 (NOX2)‐reactive oxygen species (ROS)‐nitric oxide (NO)‐myeloperoxidase (MPO) axis (Chacon et al. 2024), creating a self‐reinforcing feedback cycle between NETs and Th2 inflammation. Our study specifically examines neutrophilic inflammation in allergic asthma induced by ovalbumin (OVA) sensitization, rather than in the distinct Th2‐low neutrophilic asthma subtype, which is associated with severe symptoms, poor corticosteroid responsiveness, and increased exacerbation risk (Kuruvilla et al. 2019; Ray and Kolls 2017).

NETs are three‐dimensional fibrous structures released by neutrophils in response to pathogens or inflammatory stimuli (Chen et al. 2022). They consist primarily of DNA scaffolds associated with histones and granular proteins (Wang et al. 2024). Clinical studies consistently link excessive NET formation to poor outcomes in chronic airway inflammation, including accelerated disease progression, increased exacerbation frequency, and reduced treatment efficacy (Radermecker et al. 2019; Toussaint et al. 2017). The interplay between NETs and Th2 immunity amplifies allergic inflammation: NET‐derived components enhance dendritic cell antigen presentation, which further drives Th2 polarization and cytokine release (IL‐4, IL‐5, IL‐13) (Radermecker et al. 2019; Toussaint et al. 2017). Experimental suppression of NETs has been shown to markedly improve asthma outcomes, reducing eosinophil counts, serum IgE levels, and mucus hypersecretion (Shen et al. 2023). Despite advances with Th2‐targeted biologics such as omalizumab and dupilumab, therapeutic options for neutrophil‐driven asthma remain limited. This gap highlights the urgent need for strategies that simultaneously target neutrophilic inflammation and Th2 responses, particularly in treatment‐resistant asthma.

Nobiletin, a polymethoxylated flavonoid derived from *Citrus reticulata* ‘Chuanju’ and *Citrus aurantium* (Rutaceae family), exhibits diverse pharmacological properties, including antitumor (Chen et al. 2024), anti‐inflammatory (Fan et al. 2023), antiallergic (Jang et al. 2013), and antioxidant (Cheng et al. 2024; Zhang et al. 2020) activities. It has been investigated in cancer, cardiovascular, and neurological diseases (Chen et al. 2023; Pang et al. 2023). Recent studies suggest that nobiletin may also alleviate asthma by suppressing airway inflammation and oxidative stress, partly through inhibition of NF‐κB and activation of Nrf2 signaling, thereby decreasing Th2 cytokines (IL‐4, IL‐5, IL‐13) (Chen et al. 2025).

The PI3K/AKT and STAT3 pathways are central regulators of inflammatory and immune processes. STAT3 activation promotes inflammation through multiple mechanisms: upregulation of IL‐6, modulation of Th17/Th2 differentiation, and facilitation of airway remodeling via smooth muscle hyperplasia and mucus hypersecretion (Jie et al. 2023). In parallel, PI3K/AKT signaling enhances asthmatic inflammation by promoting Th2 cytokine production and eosinophil recruitment (Ma et al. 2021), while the AKT/STAT3 axis drives neutrophilic inflammation and tissue injury in chronic pulmonary diseases (Qinjun et al. 2024). Although the contribution of NETs to asthma pathogenesis is increasingly recognized, effective NET‐targeted therapies remain lacking. Therefore, this study aimed to determine whether nobiletin could attenuate allergic airway inflammation by inhibiting NET formation in an IgE‐mediated murine asthma model. Furthermore, we investigated whether this effect involves suppression of STAT3 and PI3K/AKT signaling, thereby providing mechanistic insights into its dual anti‐Th2 and anti‐neutrophil activities.

## 2 Methods

### 2.1 Ethics Statement

This study was approved by the Ethics Committee of Suzhou Ninth Hospital Affiliated with Soochow University (Jiangsu, China; Approval No. KYLW2023‐055‐01). C57BL/6J mice were obtained from the Laboratory Animal Center of Soochow University and maintained under controlled conditions (21°C–23°C, 51%–65% humidity) with appropriate ventilation, 12 h light/dark cycles, and ad libitum access to food and water. All experimental procedures were conducted by trained personnel in compliance with international animal welfare guidelines.

### 2.2 Asthma Model Induction

Male C57BL/6J mice (6 weeks old) were acclimatized for 1 week before being randomly assigned into experimental groups (*n* = 6 per group) using a computer‐generated randomization scheme. Sensitization was performed by intraperitoneal injection of 200 μL antigen solution containing 20 μg OVA emulsified in 100 μL saline and 100 μL aluminum hydroxide adjuvant on days 0, 7, and 14. The OVA dosage and sensitization intervals were optimized based on previously established protocols from our laboratory (Wang et al. 2025; Wu et al. 2016). Following sensitization, the mice were challenged daily by airway exposure to 2% OVA aerosol via 30 min nebulization from days 21 to 27. The OVA concentration, nebulization time, and challenge duration were selected according to standardized protocols (Wang et al. 2025; Wu et al. 2016) to ensure consistent induction of airway hyperresponsiveness and inflammation. Control mice received parallel treatments with sterile saline.

### 2.3 In Vivo Nobiletin, LY294002, and AG490 Treatment

Thirty minutes before each nebulization session, the mice received their assigned treatments. The dexamethasone (DEX) group received 5 mg/kg DEX (200 μL, intraperitoneal injection (i.p.)); the nobiletin groups were treated with 20 mg/kg (Nob‐H), 10 mg/kg (Nob‐M), or 5 mg/kg (Nob‐L) nobiletin (200 μL, i.p.); and the LY294002 and AG490 groups were administered 10 mg/kg of each inhibitor (i.p.). DEX was selected as a positive control because, although its therapeutic efficacy is limited in pure neutrophilic asthma due to insufficient promotion of neutrophil apoptosis, it remains partially effective in OVA‐induced allergic asthma involving mixed eosinophilic and neutrophilic inflammation. In IgE/Th2‐driven models with secondary neutrophil recruitment and NET formation, DEX is considered a clinically relevant comparator for evaluating nobiletin's anti‐inflammatory effects (Wang et al. 2016). Twenty‐four hours after the final challenge, the mice were anesthetized with sodium pentobarbital (50 mg/kg, i.p.) and euthanized by cervical dislocation. Bronchoalveolar lavage fluid (BALF), lung tissue, and blood samples were collected and processed immediately according to established laboratory protocols.

### 2.4 Reagents and Antibodies

All key experimental reagents, including OVA, adjuvants, pharmacological inhibitors, ELISA kits, PCR reagents, flow cytometry antibodies, tissue dissociation enzymes, and primary antibodies for immunohistochemistry and signaling pathway analyses, were purchased from commercial suppliers. Detailed information on catalog numbers, manufacturers, and applications is provided in Table S1.

### 2.5 Histopathological Evaluation

Airway inflammation was evaluated in tracheal and lung tissue sections using hematoxylin and eosin (H&E) staining. The sections were randomly selected and scored in a blinded manner (Dong et al. 2021) using a five‐tier grading system: 1 (no inflammatory cells), 2 (occasional inflammatory cells), 3 (1–3 cell layers surrounding bronchi), 4 (4–5 cell layers surrounding bronchi/vessels), and 5 (> 5 cell layers). For each animal, five randomly selected non‐overlapping fields at 100× magnification were analyzed. Mucus secretion was assessed with periodic acid–Schiff (PAS) staining, and PAS‐positive areas were quantified using Image‐Pro Plus software (version 6.0). The percentage of positively stained epithelium was calculated from at least five bronchioles per sample (Qu et al. 2017). Images were acquired at 200× magnification, and PAS‐positive areas were expressed as a percentage of the total bronchial epithelial area. Collagen deposition was assessed by Masson's trichrome staining and quantified as the percentage of positively stained area relative to the total tissue area (Dong et al. 2021; Qu et al. 2017). Image analysis was performed on five randomly selected bronchioles or parenchymal regions per sample using Image‐Pro Plus software (Media Cybernetics, Silver Spring, MD, USA). All histological assessments were performed independently by two blinded observers.

### 2.6 IgE, MPO, and NET Measurements

For sample collection, the mice were anesthetized with sodium pentobarbital (50 mg/kg, i.p.) to ensure deep anesthesia. Blood samples were collected, allowed to clot at room temperature for 1 h, and centrifuged (1000 × g, 20 min, 4°C). Serum was aliquoted and stored at −80°C until analysis. Serum total IgE concentrations and NET levels were determined using commercial ELISA kits according to the manufacturers' protocols. MPO activity was measured with standardized colorimetric assays. For BALF collection, the trachea was exposed, and an intratracheal cannula was inserted via the cricoid cartilage. A total of 0.8 mL sterile saline was instilled, allowed to dwell for 30 s, and gently withdrawn. This lavage was repeated three times, yielding ~0.7 mL BALF. The samples were centrifuged (350 × g, 10 min, 4°C), and supernatants were aliquoted and cryopreserved. NET levels in BALF were measured by ELISA, and MPO activity was assessed by colorimetric assay. After lavage, the mice were euthanized with an overdose of pentobarbital sodium.

### 2.7 Flow Cytometry

The left lung was excised, rinsed with PBS, and minced for enzymatic digestion. Samples were incubated with 2.5 mL digestion buffer (1.5 mg/mL Dispase II, 1.5 mg/mL Collagenase I, 20 U/mL DNase I) for 1 h at 37°C with shaking. The digested suspension was filtered through a 40‐μm strainer and centrifuged (350 × g, 5 min), and the supernatant was discarded. Both lung tissue pellets and BALF cell pellets were treated with 1 mL of red blood cell (RBC) lysis buffer for 5 min, followed by centrifugation (350 × g), to obtain single‐cell suspensions. Single‐cell suspensions were first incubated with Fc receptor blocking reagent (5 min) to minimize nonspecific binding. Cells were then stained with fluorescently conjugated antibodies: anti‐CD45‐PE/Cy7, anti‐CD11b‐FITC, anti‐Gr‐1‐APC, and anti‐Siglec‐F‐BV421. Cell viability was assessed with 7‐AAD‐PerCP/Cy5.5 staining (5 min). Neutrophils were defined as CD45^+^CD11b^+^Gr‐1^hi^ cells, and eosinophils were defined as CD45^+^CD11b^+^Siglec‐F^+^ cells. Samples were analyzed on a BD FACS Canto II flow cytometer (BD Biosciences, San Jose, CA, USA), and data were processed using FlowJo software (FlowJo LLC, Ashland, OR, USA; version 10.8.1). Appropriate compensation controls and standardized gating strategies were applied throughout to ensure analytical rigor.

### 2.8 Quantitative Real‐Time PCR (qPCR)

Total RNA was extracted from lung tissues and cultured cells using TRIzol according to the manufacturer's protocol. cDNA was synthesized with a commercial reverse transcription kit. Quantitative PCR was performed on a Roche LightCycler 480 system using 2 × SYBR Green Master Mix. Gene‐specific primers (Table 1), including 18 s rRNA as the internal control, were synthesized commercially (Sangon Biotech, Shanghai). All reactions were run in technical triplicates. Relative gene expression was calculated using the 2^–ΔΔCt method normalized to 18 s rRNA.

**TABLE 1:** Primers used for qPCR assay.

Gene	Forward	Reverse
Il4	TGTACCAGGAGCCATATCCA	TTCTTCGTTGCTGTGAGGAC
Il5	GGCTTCCTGTCCCTACTCAT	TCCTCGCCACACTTCTCTTT
Il13	AGCATGGTATGGAGTGTGGA	TTGCAATTGGAGATGTTGGT
Il6	TAGTCCTTCCTACCCCAATTTCC	TTGGTCCTTAGCCACTCCTTC
Il17	GAGAGCTTCATCTGTGTCTCTG	GCGCCAAGGGAGTTAAAGAC
Tnf	CCCTCACACTCAGATCATCTTCT	GCTACGACGTGGGCTACAG
18 s	CGGCTACCACATCCAAGGAA	GCTGGAATTACCGCGGCT

### 2.9 Immunofluorescence

NET formation in lung tissues was assessed by tyramide signal amplification (TSA)‐based immunofluorescence on paraffin‐embedded sections. The staining protocol included sequential incubation with: (i) anti‐Cit‐H3 primary antibody, followed by FITC‐conjugated secondary antibody with TSA amplification, and (ii) anti‐NE primary antibody with appropriate secondary antibody, both performed after antigen retrieval. Nuclei were counterstained with 4′,6‐diamidino‐2‐phenylindole (DAPI). Images were acquired using a Zeiss LSM 510 confocal microscope (Carl Zeiss AG, Oberkochen, Germany). NET quantification was conducted with Image‐Pro Plus 6.0 (Media Cybernetics, Rockville, MD, USA), using manual thresholding of fluorescence intensity to identify Cit‐H3/NE/DAPI triple‐positive structures. At least five randomly selected fields per group were analyzed, and all analyses were performed in a blinded manner.

### 2.10 Molecular Docking Analysis

The three‐dimensional structure of nobiletin (CID: 72344) was obtained from PubChem in structure‐data file (SDF) format. Crystal structures of PI3K (PDB ID: 4 L23) and STAT3 (PDB ID: 6NJS) were downloaded from the Protein Data Bank. Protein preparation, including removal of water molecules and addition of polar hydrogens, was performed using PyMOL (v2.5.2, Schrödinger). Nobiletin was energy‐minimized in ChemBio3D Ultra 14.0 (PerkinElmer) using the MMFF94 force field. Docking grids were generated in AutoDock Tools (v1.5.6), encompassing the active sites of the target proteins. Docking simulations were carried out with AutoDock Vina (v1.1.2) using an exhaustiveness parameter of 20. The 10 best binding poses were ranked by predicted binding affinity (kcal/mol). The most favorable docking conformation was visualized in PyMOL, and specific intermolecular interactions were analyzed in detail.

### 2.11 Western Blotting

Mouse lung tissue samples (50 mg) were homogenized in radioimmunoprecipitation assay (RIPA) buffer, and supernatants were collected by centrifugation. Protein concentrations were determined using the bicinchoninic acid (BCA) assay and normalized across samples. Equal amounts of protein were mixed with loading buffer, denatured by boiling for 10 min, separated by SDS‐PAGE, and transferred onto polyvinylidene fluoride (PVDF) membranes. The membranes were blocked with 5% skim milk for 2 h at room temperature, incubated overnight at 4°C with primary antibodies at appropriate dilutions, and then incubated for 2 h with HRP‐conjugated secondary antibodies. Protein bands were visualized using enhanced chemiluminescence (ECL) substrate.

### 2.12 Statistical Analysis

All data were initially assessed for normality using the Shapiro–Wilk test and for homogeneity of variance using Levene's test. Normally distributed data are expressed as mean ± SD, while non‐normally distributed data are expressed as median (IQR). For datasets meeting both assumptions, one‐way ANOVA was performed, followed by Dunnett's post hoc test for comparisons against the control group. For datasets that did not satisfy normality assumptions, the nonparametric Kruskal–Wallis test was used, followed by Dunn's post hoc test. Statistical analyses were performed using GraphPad Prism 8.0 (GraphPad Software, San Diego, CA, USA), and *p* < 0.05 was considered statistically significant.

## 3 Results

### 3.1 Effects of Nobiletin on Allergic Asthma‐Associated Inflammation, Mucus Hypersecretion, and Collagen Deposition

**FIGURE 1:**
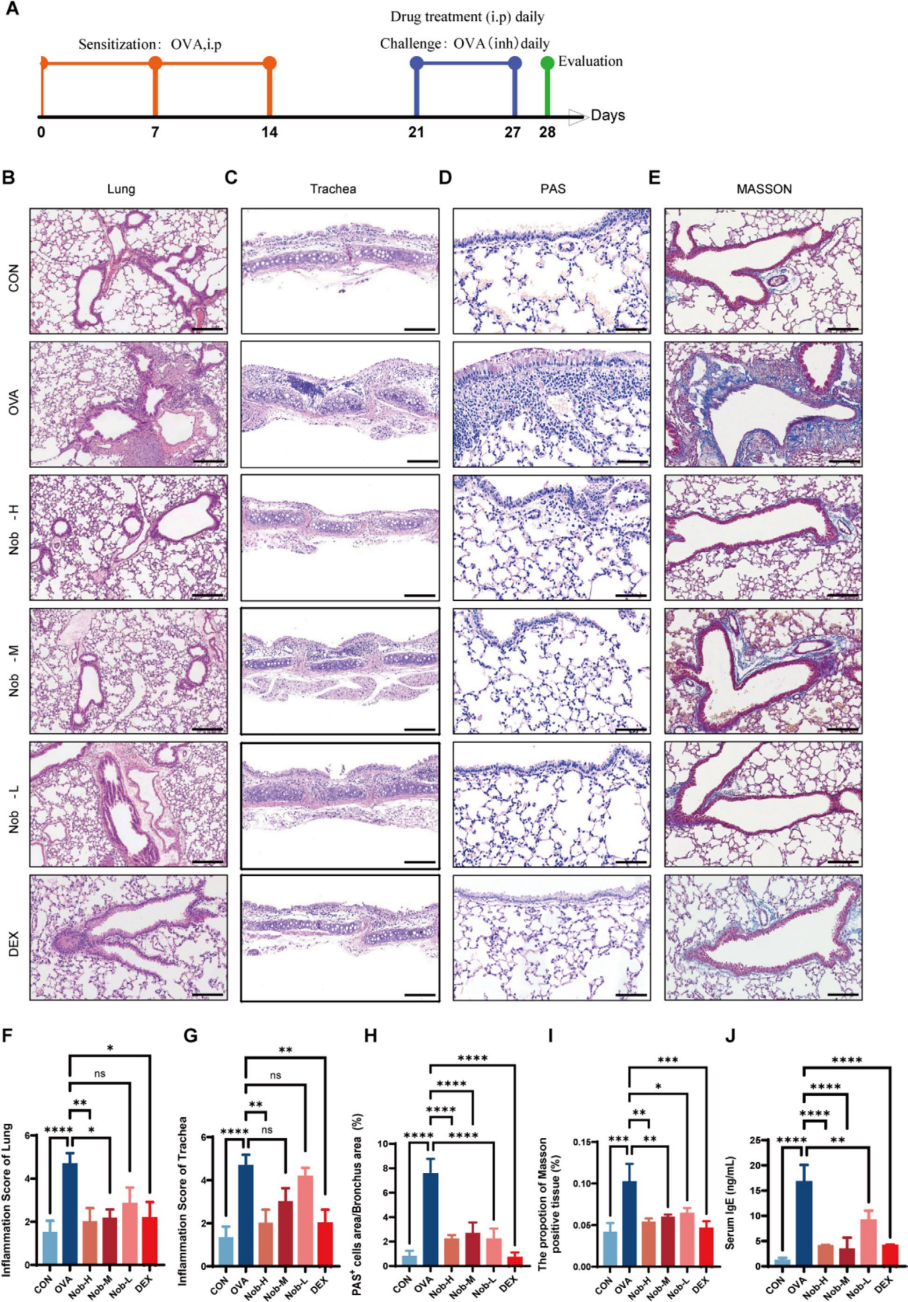
Nobiletin attenuates OVA‐induced allergic asthma via suppressing airway inflammation, mucus production, and fibrosis in a dose‐dependent manner. (A) Schematic diagram of the OVA‐induced allergic asthma model and pharmacological intervention protocol. (B) H&E staining of lung tissues (100× magnification) and (C) tracheal sections (100× magnification) collected after the final nebulization (scale bar: 100 μm). (D) PAS staining of lung sections (goblet cell metaplasia, 200× magnification) and (E) Masson's trichrome staining (collagen deposition, 200× magnification) (scale bar: 50 μm). (F) Quantitative inflammation score of lung H&E staining (*n* = 6 biologically independent samples). (G) Quantitative inflammation score of tracheal H&E staining (*n* = 6 biologically independent samples). (H) PAS staining score of lung tissues (*n* = 6 biologically independent samples). (I) Quantitative analysis of Masson's trichrome‐positive area in lung sections (*n* = 6 biologically independent samples). (J) Serum IgE levels measured by ELISA 24 h after the final nebulization (*n* = 3 biologically independent samples). Data are presented as mean ± SD. ns: Not significant; *, *p* < 0.05; **, *p* < 0.01; ***, *p* < 0.001; ****, *p* < 0.0001 vs. OVA group (one‐way ANOVA with Dunnett's post hoc test, Kruskal–Wallis test with Dunn's post hoc test).

The experimental schedule for OVA‐induced asthma and pharmacological interventions is shown in Figure 1A. To assess the effects of nobiletin on airway pathology, lung and tracheal tissues were analyzed by H&E, Masson's trichrome, and PAS staining, with blinded pathological scoring according to established criteria (Dong et al. 2021; Qu et al. 2017). In OVA‐challenged mice, histological analysis revealed extensive peribronchial and perivascular inflammatory cell infiltration, epithelial damage, luminal narrowing, and alveolar destruction (Figure 1B,C). Nobiletin treatment significantly alleviated these pathological changes in a dose‐dependent manner. The high‐dose group (Nob‐H) exhibited inflammation scores similar to those of the DEX group (*p <* 0.01 vs. OVA; Figure 1F,G). PAS staining demonstrated reduced goblet cell metaplasia and mucus hypersecretion across all nobiletin‐treated groups (*p <* 0.05; Figure 1D,H), with the strongest suppression in Nob‐H. Likewise, Masson's trichrome staining revealed significantly less subepithelial collagen deposition following nobiletin treatment (*p <* 0.05 for Nob‐L; *p <* 0.01 for Nob‐M and Nob‐H; Figure 1E,I). ELISA confirmed a dose‐dependent reduction in serum IgE (*p <* 0.01 for Nob‐M and Nob‐H; Figure 1J). Quantitative analyses across all markers demonstrated a clear dose–response relationship, with Nob‐H consistently outperforming Nob‐M and Nob‐L. Nonetheless, both Nob‐M and Nob‐L also conferred significant improvement compared with OVA alone (*p <* 0.05).

### 3.2 Nobiletin Treatment Reduces Eosinophil and Neutrophil Infiltration in BALF and Lung Tissues

**FIGURE 2:**
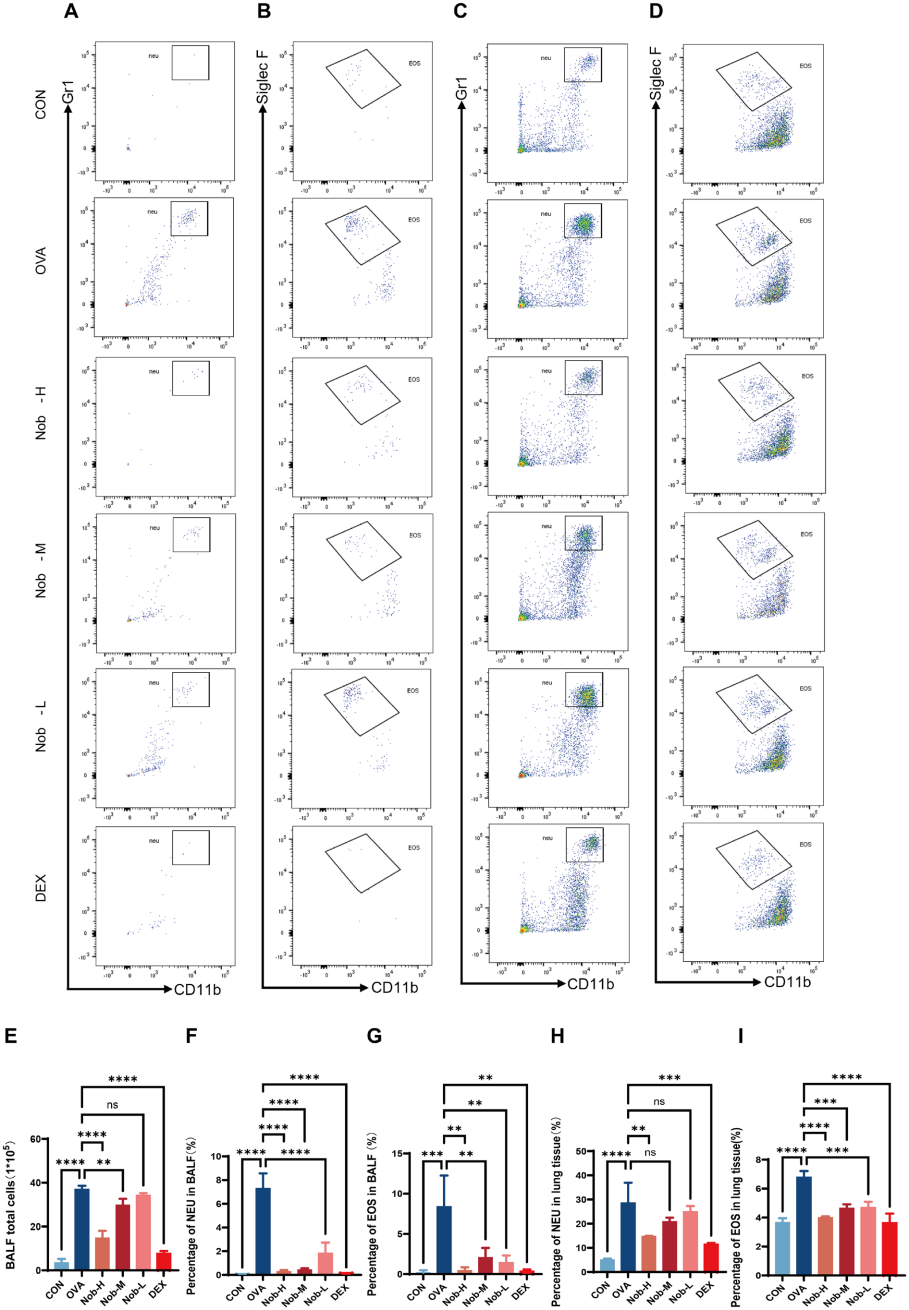
Nobiletin attenuates OVA‐induced eosinophil and neutrophil infiltration in BALF and lung tissues of asthmatic mice. (A) Representative flow cytometry plots of BALF neutrophils (CD45^+^CD11b^+^Gr1^+^). (B) Representative flow cytometry plots of BALF eosinophils (CD45^+^CD11b^+^SIglec‐F^+^). (C) Representative flow cytometry plots of lung tissues neutrophils (CD45^+^CD11b^+^Gr1^+^). (D) Representative flow cytometry plots of lung tissues eosinophils (CD45^+^CD11b^+^SIglec‐F^+^). (E) Total cell counts in BALF. (F) Neutrophil percentages in BALF. (G) Eosinophil percentages in BALF. (H) Neutrophil percentages in lung tissues. (I) Eosinophil percentages in lung tissues. Data are presented as mean ± SD (*n* = 3 per group). ns: Not significant, **, *p* < 0.01; ***, *p* < 0.001; ****, *p* < 0.0001 vs. OVA group (one‐way ANOVA with Dunnett's post hoc test).

Flow cytometry and hemocytometer analysis revealed extensive inflammatory cell infiltration in OVA‐challenged mice. BALF neutrophil and eosinophil proportions were significantly elevated following OVA challenge but reduced by nobiletin treatment. Specifically, neutrophil levels in BALF were significantly decreased in all nobiletin groups compared with OVA (*p <* 0.0001 for Nob‐L, Nob‐M, and Nob‐H; Figure 2B), while eosinophil percentages were also significantly reduced (*p <* 0.01; Figure 2A). In lung tissues, eosinophil infiltration was reduced by nobiletin in a dose‐dependent manner (*p <* 0.0001 for Nob‐H; *p <* 0.001 for Nob‐M and Nob‐L vs. OVA; Figure 2F). However, only Nob‐H significantly decreased pulmonary neutrophils compared with OVA (*p <* 0.01; Figure 2G), whereas Nob‐L and Nob‐M showed no significant effect. Total BALF cell counts were elevated in OVA‐challenged mice and suppressed by all doses of nobiletin, with Nob‐H producing the greatest reduction (Figure 2E).

### 3.3 Th2 Cytokine and Proinflammatory Mediator Expression in Lung Tissues After Nobiletin Treatment

**FIGURE 3:**
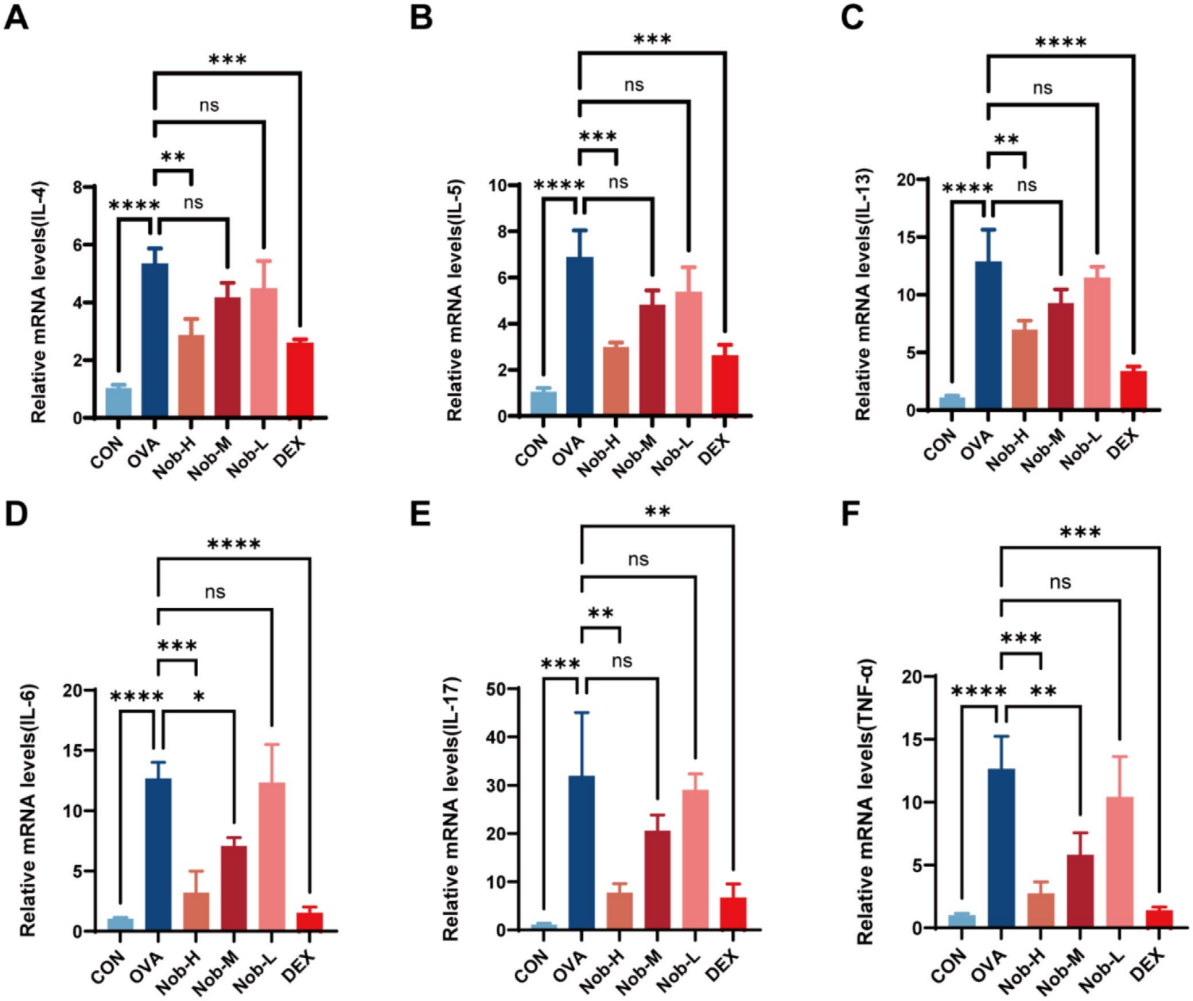
Nobiletin dose‐dependently suppresses OVA‐induced inflammatory cytokine expression in lung tissues. (A–C) mRNA expression of type 2 cytokines (IL‐4, IL‐5, IL‐13). (D–F) mRNA expression of neutrophil‐associated cytokines (IL‐6, IL‐17A, TNF‐α). Data are presented as mean ± SD (*n* = 3 per group). ns: Not significant; *, *p* < 0.05; **, *p* < 0.01; ***, *p* < 0.001; ****, *p* < 0.0001 vs. OVA group (one‐way ANOVA with Dunnett's post hoc test).

qPCR analysis revealed that OVA sensitization markedly increased the mRNA expression of Th2 cytokine genes (Il4, Il5, Il13) and neutrophil‐associated proinflammatory mediators (Il6, Il17a, Tnf) in lung tissues. Nobiletin treatment attenuated this inflammatory response in a dose‐dependent manner, with the high‐dose group (Nob‐H) producing effects largely comparable to those of DEX. Specifically, Il4, Il5, and Il13 transcript levels were significantly reduced only in Nob‐H (*p <* 0.01, < 0.001, and < 0.01, respectively, vs. OVA; Figure 3A–3C), whereas Nob‐M and Nob‐L groups did not achieve statistical significance. DEX strongly suppressed *Il4* and *Il5* (*p <* 0.001) as well as *Il13* (*p <* 0.0001). The magnitude of inhibition in Nob‐H closely mirrored that of DEX for Il5 and Il13. For neutrophil‐related cytokines, Il6 expression was significantly reduced by Nob‐H (*p <* 0.001) and Nob‐M (*p <* 0.05), with no detectable change in Nob‐L (Figure 3D). DEX showed even stronger suppression (*p <* 0.0001). Il17a expression was significantly downregulated only in Nob‐H (*p <* 0.01), with inhibition comparable to DEX (*p <* 0.01; Figure 3E). Tnf levels were significantly decreased in both Nob‐H (*p <* 0.001) and Nob‐M (*p <* 0.01), while DEX again exhibited robust suppression (*p <* 0.001; Figure 3F).

### 3.4 Quantitative Analyses of NET Formation in Pulmonary Tissues

**FIGURE 4:**
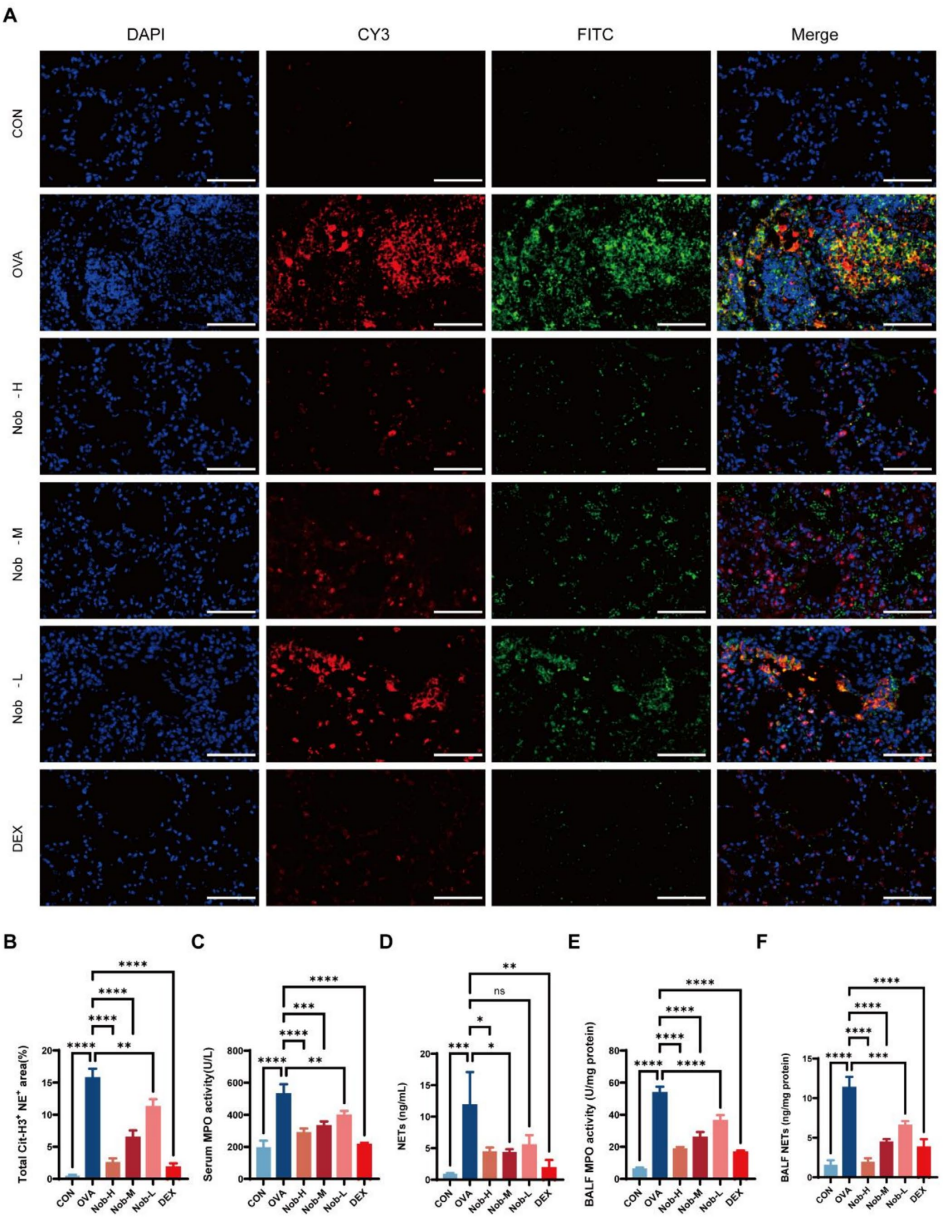
Nobiletin suppresses OVA‐induced NET formation. (A) Representative immunofluorescence images of lung sections stained for NET markers (Cit‐H3, red; NE, green; DAPI, blue) at 200× magnification (Scale bar: 50 μm). (B) Quantitative analysis of Cit‐H3^+^/NE^+^ double‐positive areas (*n* = 5 fields per group). (C) MPO activity in serum. (D) MPO activity in BALF. (E) NET levels in serum. (F) NET levels in BALF. Data are presented as mean ± SD (*n* = 3 biologically independent samples). ns: Not significant; *, *p* < 0.05; **, *p* < 0.01; ***, *p* < 0.001; ***, *p* < 0.0001 vs. OVA group (one‐way ANOVA with Dunnett's post hoc test).

OVA challenge induced pronounced pulmonary NET formation, indicated by increased Cit‐H3^+^/NE^+^ double‐positive staining in lung sections. Nobiletin administration significantly reduced NET deposition in a dose‐dependent manner (*p <* 0.01 for Nob‐L; *p <* 0.0001 for Nob‐M and Nob‐H vs. OVA), with Nob‐H achieving suppression comparable to DEX (*p <* 0.0001; Figure 4A,B). Biochemical validation supported these observations. Serum MPO activity was significantly reduced in all nobiletin‐treated groups (*p <* 0.01 for Nob‐L; *p <* 0.001 for Nob‐M; *p <* 0.0001 for Nob‐H), with Nob‐H showing an effect equivalent to DEX (*p <* 0.0001 vs. OVA; Figure 4C). Serum NET levels were also significantly decreased by Nob‐M and Nob‐H (*p* < 0.05), with a stronger effect observed in DEX‐treated mice (*p <* 0.01), whereas Nob‐L exhibited no significant change (Figure 4D). In BALF, OVA challenge markedly increased MPO activity and NET levels, both of which were significantly reduced across all treatment groups. BALF MPO activity was suppressed by all doses of nobiletin and by DEX (*p <* 0.0001 for all vs. OVA; Figure 4E). Similarly, NET levels in BALF were significantly reduced by Nob‐L (*p <* 0.001), and this effect was even more pronounced in response to Nob‐M, Nob‐H, and DEX (*p <* 0.0001; Figure 4F).

### 3.5 Association Between STAT3/PI3K‐AKT Pathway Inhibition and NET Suppression by Nobiletin

**FIGURE 5:**
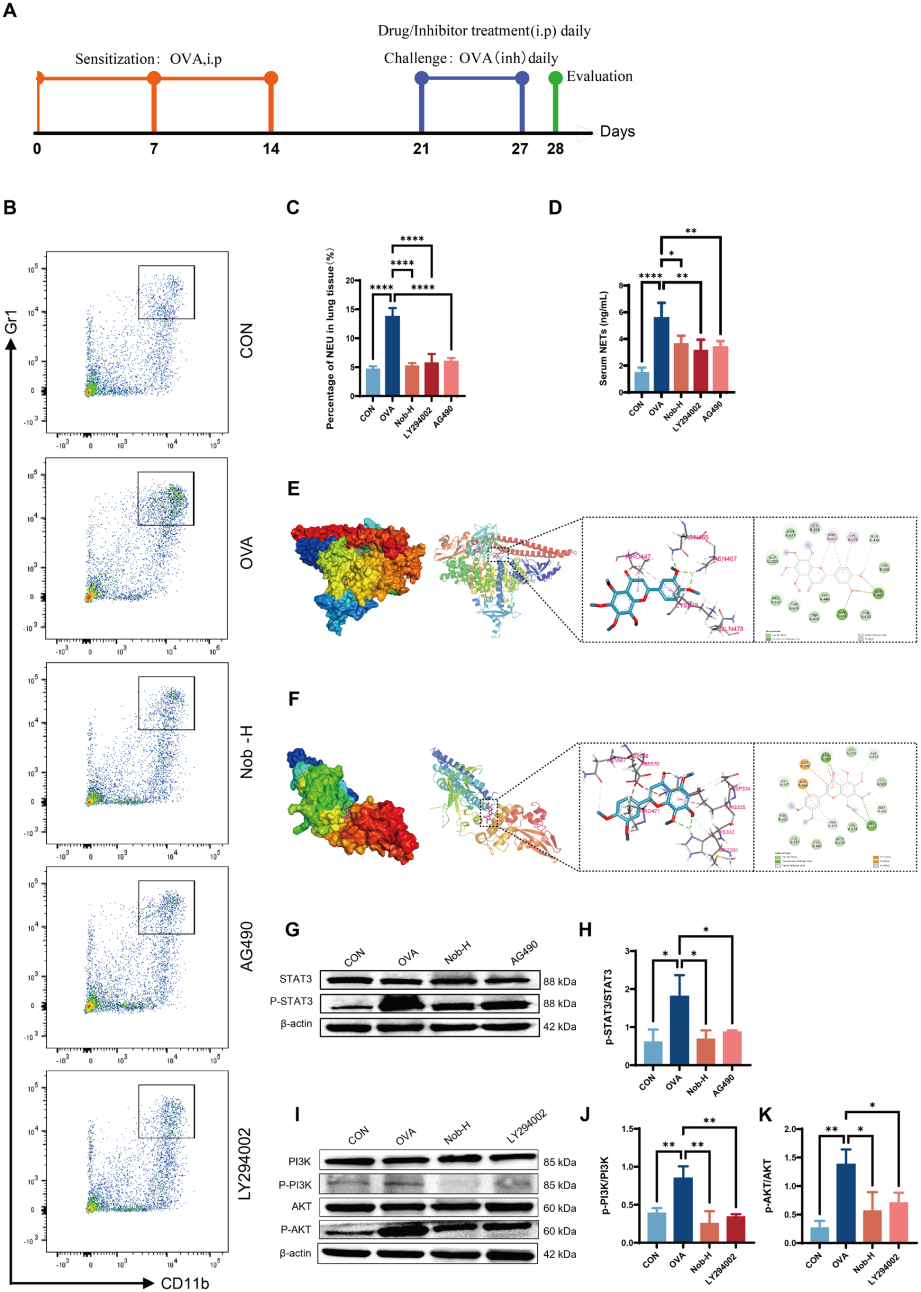
Nobiletin attenuates OVA‐induced NETosis via dual inhibition of STAT3 and PI3K/AKT pathways. (A) Experimental timeline of OVA‐induced asthma model and pharmacological interventions. (B) Flow cytometry analysis of neutrophil infiltration in lung tissues (CD45^+^CD11b^+^Gr1^+^ cells). (C) Quantification of pulmonary neutrophils. (D) Serum NET concentrations measured by ELISA. (E) Molecular docking analysis of nobiletin‐STAT3 binding affinity. (F) Molecular docking analysis of nobiletin‐PI3K binding affinity. (G) Representative western blots of p‐STAT3, total STAT3. (H) Ratio of p‐STAT3 to total STAT3 normalized to β‐Actin. (I) Representative western blots of p‐PI3K, total PI3K, p‐AKT, and AKT. (J) Ratio of p‐PI3K to total PI3K normalized to β‐Actin. (K) Ratio of p‐PI3K to total PI3K normalized to β‐Actin. (*n* = 3 independent experiments). Data are presented as mean ± SD. *, *p* < 0.05; **, *p* < 0.01; ***, *p* < 0.001; ****, *p* < 0.0001 vs. OVA group (one‐way ANOVA with Dunnett's post hoc test).

Previous studies have shown that nobiletin exerts anti‐inflammatory effects via modulation of the STAT3 and PI3K/AKT signaling pathways (Xie et al. 2019; Yin et al. 2023), prompting us to evaluate whether its inhibition of OVA‐induced NET formation is mediated through these mechanisms. Following the experimental timeline (Figure 5A), OVA‐induced asthmatic mice were treated with Nob‐H, AG490 (a STAT3 inhibitor), or LY294002 (a PI3K/AKT inhibitor). Flow cytometry demonstrated that pulmonary neutrophil infiltration was significantly reduced in the Nob‐H, LY294002, and AG490 groups (*p <* 0.0001 vs. OVA), with neutrophil levels restored to values comparable to controls (Figure 5B,C). Serum NET concentrations were likewise significantly reduced by Nob‐H (*p <* 0.05), LY294002, and AG490 (*p <* 0.01 vs. OVA; Figure 5D). Molecular docking analysis revealed the strong binding affinity of nobiletin to STAT3 (−7.6 kcal/mol; Figure 5E) and PI3K (−6.1 kcal/mol; Figure 5F), supporting its potential direct interaction with these proteins. Western blotting further confirmed that Nob‐H significantly inhibited STAT3 phosphorylation (*p <* 0.01), with effects comparable to AG490 (*p <* 0.05; Figure 5G,H). Similarly, PI3K activation (p‐PI3K/PI3K) was significantly reduced in both the Nob‐H and LY294002 groups (*p <* 0.01; Figure 5I,J). AKT phosphorylation (p‐AKT/AKT) was suppressed even more strongly by Nob‐H (*p <* 0.001) than by LY294002 (*p <* 0.01; Figure 5I,K).

## 4 Discussion

Bronchial asthma is a heterogeneous chronic airway inflammatory disease characterized by airway hyperresponsiveness and reversible airflow limitation (Wan et al. 2023). In recent years, global climate change and worsening air pollution have contributed to the rising prevalence of asthma (Yasaratne et al. 2023). Although inhaled corticosteroids (ICSs) in combination with long‐acting *β*_2_‐agonists (LABAs) remain the mainstay of therapy, alternative strategies are urgently needed for patients with suboptimal responses. Nobiletin, a polymethoxylated flavonoid predominantly derived from citrus peels (e.g., tangerines and oranges) and commercially available as a dietary supplement, exerts anti‐inflammatory effects through multi‐target and multi‐pathway regulation. First, it inhibits NF‐κB nuclear translocation, thereby downregulating the transcription of proinflammatory cytokines such as TNF‐α, IL‐6, and IL‐1β (Yue et al. 2023). Second, it suppresses CCL2 and CXCL8 expression, reducing neutrophil and monocyte chemotaxis and subsequent inflammatory mediator release (Hagenlocher et al. 2017). Third, it attenuates COX‐2 expression, thereby reducing PGE_2_ synthesis and downstream inflammatory enzyme activity (Murata et al. 2023). Additionally, Wu et al. demonstrated that nobiletin mitigates eosinophilic airway infiltration and enhances eosinophil apoptosis through upregulation of *Fas* mRNA expression (Wu et al. 2006). Taken together, these findings indicate that nobiletin may represent a promising therapeutic candidate for alleviating airway inflammation and oxidative stress in asthma, with a favorable safety profile at physiologically relevant doses.

Allergic asthma is pathologically characterized by predominant eosinophilic infiltration, and it typically demonstrates good responsiveness to corticosteroid therapy. In contrast, neutrophilic asthma is marked by elevated airway neutrophils, and it exhibits poor corticosteroid sensitivity. Increasing evidence suggests that neutrophilic infiltration may coexist within the airway microenvironment even in classical allergic asthma. The mechanisms underlying this phenomenon include the following: (1) allergen‐derived proteases activating protease‐activated receptor 2 (PAR2) on airway epithelial cells, which in turn induce chemokine release (e.g., IL‐8) and promote neutrophil recruitment (Matos et al. 2022); and (2) infiltrating neutrophils secreting IL‐6 and TGF‐β, which drive dendritic cell‐mediated Th2 differentiation and perpetuate allergic responses (Elkoshi 2024). In our OVA‐induced allergic asthma model, we observed not only significant eosinophilia but also concurrent neutrophil elevation. These findings highlight the complex interplay between neutrophils and Th2 responses in allergic airway inflammation. Nobiletin treatment significantly attenuated both classical asthmatic manifestations and neutrophilic inflammation, as evidenced by reduced neutrophil counts in BALF and lung tissues, together with decreased expression of pro‐inflammatory cytokines (TNF‐α, IL‐6, and IL‐17A). These results demonstrate nobiletin's dual regulatory capacity in allergic asthma. Our study extends previous findings by confirming the role of NETs in allergic asthma, as previously reported by (Radermecker et al. 2019) and (Toussaint et al. 2017), while also demonstrating that NETs can be suppressed through dual blockade of the STAT3 and PI3K/AKT pathways by nobiletin. This dual inhibition produced more pronounced reductions in airway inflammation compared to earlier studies targeting single pathways (e.g., DNase I or JAK inhibitors) (Kok et al. 2009; Shen et al. 2023). Unlike DNase I, which degrades pre‐formed NETs, nobiletin acts upstream by preventing NET release, thereby offering more durable immune modulation with potentially fewer adverse effects. These findings are consistent with recent evidence suggesting that combinatorial pathway inhibition may be necessary to disrupt the self‐reinforcing NET–Th2 axis (Vogt and Hart 2011). Together, our results not only corroborate prior research but also expand on it by identifying a promising mechanism‐based therapeutic strategy.

NETs are web‐like structures released during neutrophil activation that engage in complex bidirectional crosstalk with Th2 inflammation in the context of asthma pathogenesis. Mechanistically, nobiletin‐mediated NET suppression appears to be closely associated with its ability to simultaneously target STAT3 and PI3K/AKT signaling, both of which are essential for neutrophil activation and survival. STAT3 regulates Th2 cytokine transcription and upregulates NOX2 components, contributing to ROS generation (Chacon et al. 2024; Qinjun et al. 2024). In parallel, PI3K/AKT activation is required for MPO translocation and degranulation during NETosis. Dual inhibition by nobiletin interrupts this axis at both transcriptional and post‐translational levels. Furthermore, molecular docking confirmed direct binding of nobiletin to STAT3 and PI3K, reinforcing its mechanistic specificity and potency. Importantly, NETs amplify Th2 responses through multiple mechanisms. First, NET components such as Cit‐H3 and dsDNA serve as autoantigens presented by dendritic cells via MHC‐II, thereby promoting Th2 differentiation (Chopp et al. 2023). In addition, Cit‐H3 upregulates dendritic cell co‐stimulatory molecules (CD80/CD86) and induces thymic stromal lymphopoietin production, which together enhance Th2 activation (Jia et al. 2023). Neutrophil elastase further stimulates epithelial release of IL‐33, IL‐25, and TSLP, which subsequently activate Th2 cells and trigger cytokine secretion (IL‐4, IL‐5, IL‐13) (Mincham et al. 2024). Moreover, myeloperoxidase within NETs modifies extracellular matrix components, enhancing IL‐33 bioactivity and further amplifying Th2 inflammation (Liu et al. 2025). Conversely, Th2 cytokines reciprocally promote NETosis. IL‐4 and IL‐13 upregulate neutrophil CCR3 expression through STAT6 signaling, facilitating airway migration, while IL‐5 prolongs neutrophil survival and enhances ROS production (Mayoral Andrade et al. 2020). Clinical observations also support this interaction, with studies reporting positive correlations between serum IL‐13 and Cit‐H3 levels in patients with severe asthma, suggesting Th2‐mediated neutrophil reprogramming (Rahat and Shakya 2016). In line with these findings, our experimental data revealed concurrent elevation of Th2 cytokines (IL‐4, IL‐5, IL‐13) and NETs in OVA‐challenged mice, all of which were significantly reduced by nobiletin treatment. These results suggest that nobiletin may indirectly suppress NETosis through rebalancing Th1/Th2 responses. Compared with conventional NET‐targeting strategies such as DNase I, nobiletin's upstream modulation of immune balance provides distinct advantages, including sustained efficacy and a lower risk of immunosuppression associated with excessive NET degradation.

Importantly, our data demonstrate that nobiletin concurrently inhibits STAT3 and PI3K/AKT signaling pathways, thereby disrupting a critical crosstalk axis that drives NETosis. Previous studies have shown that STAT3 can transcriptionally upregulate PI3K regulatory subunits p50α and p55α through direct promoter binding (Kok et al. 2009). Conversely, PI3K/AKT activation has been reported to enhance STAT3 phosphorylation at Tyr705 in PI3K‐driven transformed cells, indicating the existence of a reciprocal amplification loop between these pathways (Vogt and Hart 2011). Nobiletin's dual‐inhibitory mode of action therefore interrupts this positive feedback loop, producing stronger suppression of NETs compared with single‐pathway targeting. This mechanistic synergy is further supported by molecular docking studies. Nobiletin was predicted to bind with high affinity to the SH2 domain of STAT3 and to the ATP‐binding pocket of PI3K, directly impeding their catalytic activities. The predicted interactions exhibited strong biological relevance for NET regulation. When interacting with STAT3, nobiletin was found to engage several residues within the DNA‐binding and SH2 domains, including Pro‐447, Asn‐465, Asn‐467, Gln‐478, and Lys‐678. These interactions may interfere with STAT3 conformational flexibility, destabilize SH2 domain architecture, or stabilize an inactive conformation, thereby preventing Tyr705 phosphorylation. Unlike AG490, a well‐characterized upstream JAK2 inhibitor that indirectly blocks STAT3 activation by inhibiting the kinase responsible for Tyr705 phosphorylation (Meydan et al. 1996), nobiletin appears to directly target STAT3. This direct inhibition may confer dual effects by impairing STAT3's phosphorylation readiness and blocking downstream dimerization events. When interacting with PI3K, nobiletin engages multiple key residues within and adjacent to the ATP‐binding pocket, including Pro‐471, Asn‐567, Asp‐566, Asp‐570, Asp‐334, Arg‐335, His‐332, and Met‐331. These residues are critical for ATP coordination and catalytic activity, with Asp‐566, Asp‐570, and Asp‐334 forming part of the conserved catalytic motif, while Arg‐335 and His‐332 stabilize substrate binding via hydrogen bonding and electrostatic interactions. This binding profile partially overlapped with that of LY294002, a known PI3K inhibitor that competitively targets the ATP‐binding cleft, primarily forming hydrophobic and hydrogen bonds with residues such as Val‐851 and Lys‐802 (Walker et al. 1999). However, unlike LY294002, nobiletin also interacted with proximal residues such as Pro‐471 and Asn‐567, potentially broadening its binding footprint and enhancing conformational modulation. This extended interaction network may contribute to more effective inhibition of PI3K and AKT activity. This interference disrupts the IgE‐NOX2‐ROS‐NO‐MPO axis (Chacon et al. 2024). Specifically, STAT3 inhibition suppresses NOX2 transcription, while PI3K blockade prevents AKT‐dependent MPO translocation, collectively impairing both ROS generation (NET initiation) and Cit‐H3/NE release (NET execution). These effects were substantiated by reductions in MPO activity and Cit‐H3^+^/NE^+^ NETs.

Unlike Th2‐focused biologics (e.g., omalizumab or dupilumab), which have limited efficacy in neutrophilic asthma, nobiletin targets both arms of allergic inflammation: (1) Th2 cytokine activity, via STAT3‐mediated suppression of GATA3 expression, and (2) neutrophil/IL‐17A‐driven pathology, through PI3K/AKT inhibition. This dual action interrupts the self‐amplifying NET‐Th2 cycle, positioning nobiletin as a promising adjunctive therapy for biologics‐resistant asthma with mixed granulocytic infiltration. Beyond acute inflammation, nobiletin's suppression of NETs may also contribute to its anti‐remodeling effects. NET‐associated proteases, including NE and MMP9, degrade extracellular matrix components (Hurt et al. 2017; Mondal et al. 2020) and activate TGF‐*β*, thereby promoting fibroblast proliferation and subepithelial fibrosis (Mousset et al. 2023). By limiting NET release, nobiletin may attenuate MMP9 activity and TGF‐β activation. These mechanisms warrant further investigation in chronic asthma models to evaluate long‐term effects on airway remodeling.

**FIGURE 6:**
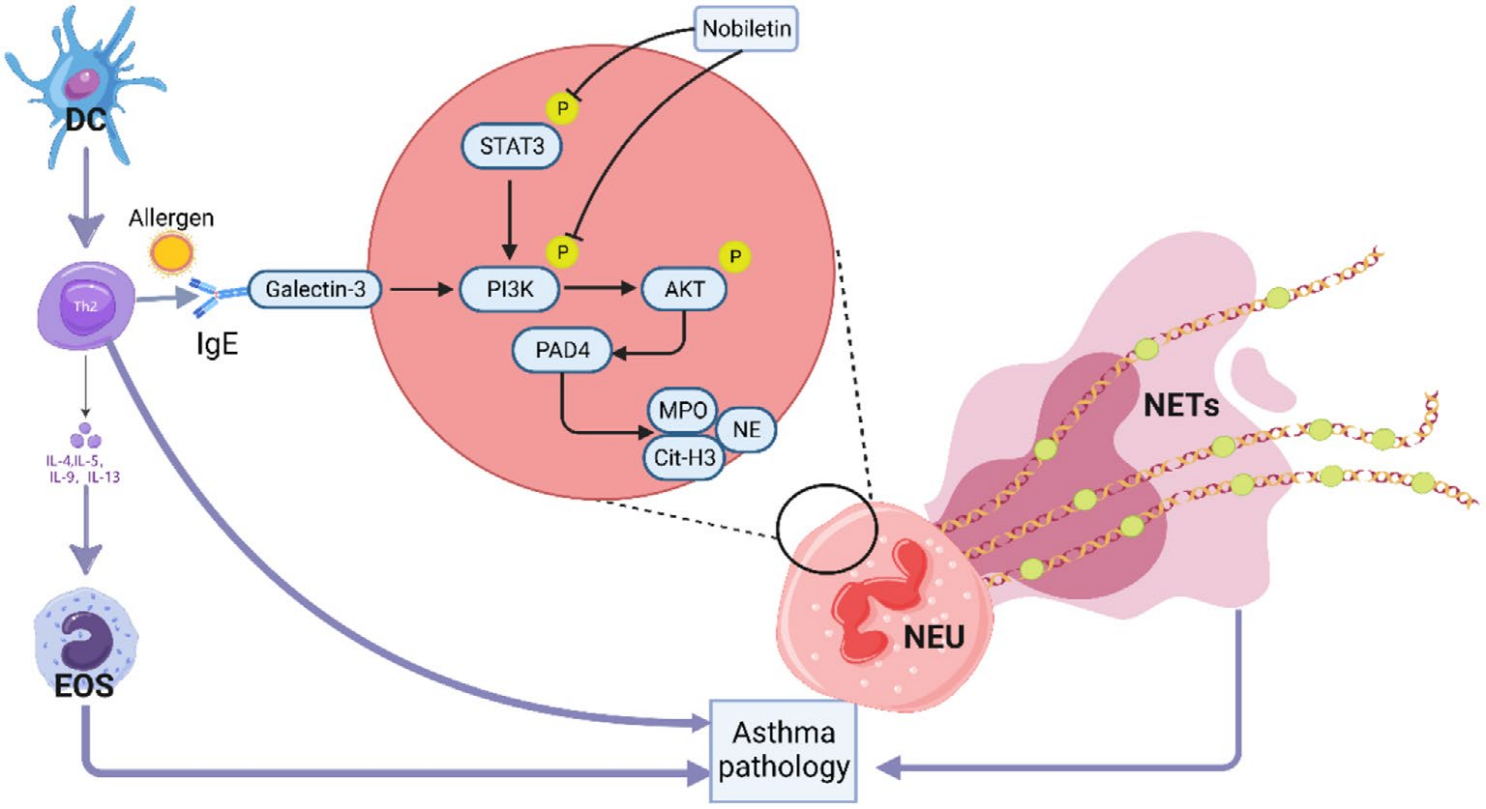
Schematic summary of nobiletin's dual‐pathway inhibitory mechanism in neutrophilic allergic asthma.

In summary, this study reveals the concomitant presence of neutrophilic infiltration within the airway microenvironment of allergic asthma. Nobiletin exhibits a dual therapeutic mechanism that surpasses conventional single‐pathway inhibitors by simultaneously: (i) reducing eosinophil/neutrophil infiltration and NET formation via suppression of PI3K/AKT and STAT3 signaling, and (ii) preventing NET‐mediated exacerbation of allergic asthma pathology (Figure 6). These findings not only advance current understanding of NET‐driven pathology but also provide a mechanistic rationale for developing nobiletin‐based therapies targeting treatment‐refractory neutrophilic asthma.

## 5 Strengths and Limitations

The major strengths of this study include the use of a well‐established OVA‐induced allergic asthma model that displays both eosinophilic and neutrophilic infiltration, closely mimicking mixed‐granulocytic phenotypes observed clinically. In addition, we employed a multi‐method approach incorporating histology, flow cytometry, molecular docking, and signaling analyses to comprehensively evaluate therapeutic mechanisms. However, several limitations should be noted. First, peripheral blood neutrophil counts were not assessed, as our primary focus was on characterizing local airway inflammation using BALF cellular profiles and lung histopathology, which were deemed most relevant to the study objectives. Nonetheless, parallel evaluation of circulating neutrophils could provide valuable insight into systemic versus local inflammatory responses. Future studies should incorporate peripheral blood analyses to strengthen phenotypic characterization. Second, the experimental design was limited to a single animal model and dosing regimen, which may not fully capture the heterogeneity of asthma phenotypes observed clinically. Expanding the range of models and validating findings in additional systems will be important to confirm the broader applicability of these results.

## 6 Conclusion

Collectively, our findings demonstrate that nobiletin effectively attenuates allergic asthma–induced airway inflammation and prevents pathological exacerbation by suppressing NET formation within the airways. These results identify nobiletin as a potential NET‐targeting therapeutic candidate for the treatment of airway inflammatory diseases.

## Author Contributions

**Wen He:** conceptualization (equal), data curation (equal), formal analysis (equal), writing – original draft (equal), writing – review and editing (equal). **Yuqing Xu:** data curation (equal), formal analysis (equal), methodology (equal). **Lin Zhu:** data curation (equal), formal analysis (equal). **Juzhang Li:** data curation (equal). **Jianfeng Zhang:** methodology (equal), writing – review and editing (equal). **Qiaozhen Wu:** funding acquisition (equal), methodology (equal), supervision (equal), writing – review and editing (equal).

## Ethics Statement

All animal experiments were approved by the Ethics Committee of Suzhou Ninth Hospital Affiliated to Soochow University (Jiangsu, China; Approval No. KYLW2023‐055‐01).

## Conflicts of Interest

The authors declare no conflicts of interest.

## Supporting information


**Table S1:** Comprehensive List of Reagents and Antibodies.

## Data Availability

The data that support the findings of this study are available from the corresponding author upon reasonable request.
