# Supplementary material for: Natural Flavonoid Nobiletin Attenuates Allergic Asthma via Suppression of STAT3/PI3K‐AKT and Neutrophil Extracellular Traps
Source: Food Sci Nutr. 2025 Oct 16;13(10):e71083. doi: 10.1002/fsn3.71083 (PMC12531424; doi:10.1002/fsn3.71083)
Supplement: Supplementary file 1 — Table S1: Comprehensive List of Reagents and Antibodies. [file FSN3-13-e71083-s001.docx]

**Supplementary Table S1: Comprehensive List of Reagents and Antibodies**

| **Category** | **Specific Reagent** | **Vendor** | **Catalog Number** | | **Application/Details** |
| --- | --- | --- | --- | --- | --- |
| **Reagents** | Ovalbumin (Grade V) | Sigma-Aldrich | | A5503 | Asthma model induction |
|  | Aluminum hydroxide adjuvant | Thermo Fisher | | 77161 | OVA emulsification |
|  | Nobiletin (purity: 99.76%) | TargetMol Chemicals | | T2834 | 5/10/20 mg/kg i.p. treatment |
|  | LY294002 (purity: 99.93%) | TargetMol Chemicals | | T2008 | 10 mg/kg i.p. treatment |
|  | AG490 (purity: 99.39%) | TargetMol Chemicals | | T2600 | 10 mg/kg i.p. treatment |
|  | Dexamethasone(purity: 99.91%) | TargetMol Chemicals | | T1076 | 5 mg/kg i.p. positive control |
| **Kits** | Mouse IgE ELISA Kit | Dakewe Biotech | | 1218202 | Serum IgE quantification |
|  | Myeloperoxidase (MPO) Assay Kit | Jiancheng Bioengineering | | A044-1-1 | MPO activity in serum/BALF |
|  | NETs Quantification Kit | Jianglai Biotechnology | | JL47089 | NET levels in serum/BALF |
|  | SYBR Green Master Mix | Vazyme Biotech | | Q712-02 | qPCR amplification |
|  | Reverse Transcription Kit | Vazyme Biotech | | R323-01 | cDNA synthesis |
| **Antibodies** | **Flow Cytometry** |  | |  |  |
|  | Anti-mouse CD45-BV421 | BioLegend | | 103134 | 1:100 dilution |
|  | Anti-mouse CD11b-BV510 | BioLegend | | 101206 | 1:100 dilution |
|  | Anti-mouse Gr-1-APC-Cy7 | BioLegend | | 108412 | 1:100 dilution |
|  | Anti-mouse Siglec-F-PE | BioLegend | | 155509 | 1:100 dilution |
|  | Fc receptor blocker | BioLegend | | 156603 | Pre-staining blocking |
|  | **Immunohistochemistry** |  | |  |  |
|  | Anti-Cit-H3 monoclonal antibody | Abcam | | ab5103 | IHC/IF (1:200) |
|  | Anti-NE monoclonal antibody | Abclonal | | A8953 | IHC/IF (1:200) |
|  | **Western Blot** |  | |  |  |
|  | Anti-STAT3 | Cell Signaling | | 9139T | WB (1:1000) |
|  | Anti-p-STAT3 | Cell Signaling | | 9145T | WB (1:1000) |
|  | Anti-PI3K | Cell Signaling | | 4257T | WB (1:1000) |
|  | Anti-p-PI3K | Cell Signaling | | 4228T | WB (1:1000) |
|  | Anti-AKT | Cell Signaling | | 4691T | WB (1:1000) |
|  | Anti-p-AKT | Cell Signaling | | 4060T | WB (1:1000) |
| **Enzymes** | Collagenase Type I | BioFroxx Scientific | | 1904mg100 | Tissue dissociation (1.5 mg/ml) |
|  | DNase I | BioFroxx Scientific | | 1121mg100 | Tissue dissociation (20 U/ml) |
|  | Hyaluronidase | BioFroxx Scientific | | 1141mg100 | Tissue dissociation  (1.5 mg/ml) |
